# How Do I Get on With my Teacher? Affective Student‐Teacher Relationships and the Religious Match Between Students and Teachers in Islamic Primary Schools

**DOI:** 10.1111/bjep.12457

**Published:** 2021-09-08

**Authors:** Fatima Zohra Charki, Lisette Hornstra, Jochem Thijs

**Affiliations:** ^1^ Department of Education Utrecht University The Netherlands; ^2^ Department of Interdisciplinary Social Science Utrecht University The Netherlands

**Keywords:** attitude, Islamic schools, Muslim children, religious incongruence, student‐teacher relationship, teachers

## Abstract

**Background:**

Despite the growing body of research concerning affective relationships between teachers and ethnic minority students, very little is known about student‐teacher relationship (STR) quality for religious minority students. Many Islamic schools have a mixed workforce consisting of both Muslim and non‐Muslim teachers. This means that the quality of religiously congruent and religiously incongruent STRs can be directly compared.

**Aims:**

We investigated whether the quality of the STR experienced by Dutch Islamic school students depended on the religious background of their teacher (Muslim vs. non‐Muslim). We also examined the role of teachers’ implicitly measured attitudes towards Muslims as a possible explanation for differences in relationship quality.

**Sample:**

Participants were 707 students (56.9% female) from 35 classes (Grade 3–6) (*M*_age_ = 10.02 years, *SD* = 1.25) and their 35 teachers (85.7% female; *M*_age_ = 32.94 years, *SD* = 6.37).

**Methods:**

Students reported on the quality of the relationship with their teacher (closeness, conflict, and negative expectations), and teachers’ implicit attitude towards Muslims (vs. non‐Muslims) was measured with an Implicit Association Test.

**Results:**

Students reported relatively high levels of closeness and low levels of conflict and negative expectations for both Muslim and non‐Muslim teachers. Conflict was slightly higher in religiously incongruent STRs, but only when teachers’ implicitly measured attitude towards Muslims (vs. non‐Muslims) was included in our model.

**Conclusion:**

Results of this study indicate that religious incongruence does not play a major role in STR quality in Islamic primary education.

## Background

The affective relationships between primary school students and their teachers have been studied with increasing interest in the last decades (Bosman, Roorda, van der Veen, & Koomen, 2018; Howes, Hamilton, & Matheson, 1994; Pianta, 1994), and there is clear evidence that students’ emotional and academic adjustment is at risk when these relationships are negative (Roorda, Koomen, Spilt, & Oort, 2011; Sabol & Pianta, 2012). Research has shown that (some groups of) ethnic or racial (ER) minority students are particularly likely to develop negative student‐teacher relationships (STRs) (Irvine & Fenwick, 2011; Naman, 2009; Spilt & Hughes, 2015; Thijs, Westhof, & Koomen, 2012). One explanation for this finding is that, due to the composition of the educational workforce, minority students often have majority teachers (Howes & Shivers, 2006; Saft & Pianta, 2001). As a result, their relationships are ER incongruent, and could therefore be compromised by intergroup biases and cultural misunderstandings (Rasheed, Brown, Doyle, & Jennings, 2019; Thijs et al., 2012).

Although some of the ER minority children studied in earlier research (e.g., Moroccan‐Dutch) also belong to religious minority groups (e.g., Muslims), there is a lack of research on STRs quality in religious minority students. In Western Europe, Muslims constitute a salient minority group that faces much prejudice and discrimination (Strabac & Listhaug, 2008), and although this group includes people from various ethnic backgrounds, its shared religious values (partly) differ from those of the non‐Muslim native majority population. Thus, Muslim children in Western Europe may also have less favourable relationships with their teachers due to group biases and ‘cultural’ misunderstandings. In this study, we examined how Muslim children in Dutch Islamic primary schools experienced the relationship with their primary teacher. Not all teachers there are Muslims themselves (Driessen & Valkenberg, 2000), and this means that the quality of religiously congruent and incongruent STRs can be directly compared. In addition, we examined the role of teachers’ implicitly measured attitudes towards Muslim children as a possible explanation for differences in relationship quality between Muslim and non‐Muslim teachers.

In the Netherlands, there is an ongoing debate about whether or not it would be beneficial for Muslim children to be taught by Muslim teachers only, with some Islamic schools striving for a mixed teaching staff consisting of both Muslim and non‐Muslim teachers, and other schools aiming for Muslim teachers only (e.g., Dronkers, 2016). This study provides insights on how congruence in teacher and student religion and implicitly measured teacher attitudes towards Muslims affect the quality of the STR in those schools. We focused on students’ rather than teachers’ perceptions of the STR, as children’s subjective relationship experiences are psychologically important for them (Pianta, Hamre, & Stuhlman, 2003). Moreover, how students themselves perceive the relationship is believed to be most relevant for their well‐being (Stewart & Suldo, 2011), motivation (Patrick, Ryan, & Kaplan, 2007), and academic achievement (Fall & Roberts, 2012).

### Student‐teacher relationships as extended attachment bonds

There is ample evidence that students’ affective relations with their teachers are crucial for their emotional well‐being, social behaviour, and school engagement (Bosman et al., 2018; Roorda et al., 2011). According to Attachment Theory (Bowlby, 1982), the quality of affective relationships between children and their primary caregivers (mostly their parents) is crucial for the way children handle stress and challenges. The so‐called extended attachment perspective (see Ainsworth, 1989) builds upon attachment theory by stating that sensitive teachers can operate as secondary attachment figures and thereby provide their students with a ‘safe base’ to engage in learning activities (Roorda et al., 2011).

Following the work of Pianta (1994), researchers working from the extended attachment perspective have examined STR quality along three dimensions: closeness, conflict, and dependency. Closeness indicates security in the STR. It reflects the extent to which student and teacher interact and communicate in a warm and positive manner, and the degree to which the student is confident about the teacher as a source of emotional support. By contrast, conflict and dependency indicate insecurity. They involve, respectively, the experience of mutually negative and distrustful feelings, and the degree to which the student is overly concerned with the teacher’s availability and in constant need of their reassurance (Pianta et al., 2003; Verschueren & Koomen, 2012). These relationship dimensions are most often assessed from the teacher’s perspective. Closeness and conflict can be reliably measured in children, but it appears more difficult to assess dependency in them (but see Thijs & Fleischmann, 2015). Koomen and Jellesma (2015) devised and tested an instrument to measure the three dimensions in children. Instead of dependency, they found another, broader dimension which they labeled ‘negative expectations’, to indicate children’s perceptions of their teacher, and more specifically, their lack of confidence in them. This dimension reflects children’s worries about their teacher’s emotional availability and responsiveness, which are typical for dependency, but also their uncertain feelings about their teacher more generally (Koomen & Jellesma, 2015). This study focuses on students’ STR perceptions using the dimensions of conflict, closeness, and negative expectations.

### Religious incongruence and STR quality

Although religion, ethnicity, and race are clearly not the same, meaningful parallels can be drawn between ER incongruence and religious STR incongruence. Prior research has shown that students can be at risk for developing unfavourable relationships with their teachers if they have different ER backgrounds, although this also seems to depend on the particular combination of backgrounds (Rasheed et al., 2019; Saft & Pianta, 2001; Thijs et al., 2012; but see Ewing & Taylor, 2009). Theoretically, there are two complementary explanations for this risk (see Thijs et al., 2012). First, according to Social Identity Theory (SIT; Tajfel & Turner, 1979), people have a basic tendency to favour the groups they belong to. As part of their identities (their social identities) is derived from these groups, this so‐called in‐group favouritism would reflect positively on their selves. Thus, based on SIT, it can be predicted that teachers and children are more positive about each other if they belong to the same group (see also Glock & Schuchart, 2020), which would enhance the quality of their relationship. Second, ER incongruent relationships could be less favourable than ER congruent ones due to misunderstandings. Different ER backgrounds often imply different cultural backgrounds. And as cultures provide shared guidelines for what to think and how to behave, differences in cultural backgrounds could result in weak understanding, poor communication, and negative interpretations of behaviour (see Van der Zee, Van Oudenhoven, & De Grijs, 2004). This in turn, could comprise the quality of the STR.

Both explanations could apply to religiously incongruent STRs as well. Religious groups can be very important for people’s identities, and they provide their members with shared rituals, traditions, and values pertaining to the correct way of life (Ysseldyk, Matheson, & Anisman, 2010). Thus, it can be expected that Muslim children will experience more closeness, less conflict, and fewer negative expectations in relationships Muslim versus non‐Muslim teachers.

### Teachers’ attitudes towards Muslims

Consistent with SIT (Tajfel & Turner, 1979), it can be further argued that the anticipated effect of religious incongruence on STR quality could be partly attributed to the teacher’s religious attitudes. That is to say, Islamic children would share comparatively more positive relationships with Muslim (vs. non‐Muslim) teachers as the latter would have a more positive attitude towards Muslims (in‐group). In this study, we tested this hypothesis by using an implicit measure for teachers’ attitudes towards Muslims versus non‐Muslims.

Attitudes can be described as evaluations of a target. Such a target can refer to a group of people (e.g., Fazio et al., 1995). Research on teacher attitudes has mostly focused on attitudes towards stigmatized groups such as ethnic minority students or special needs students (Denessen, Hornstra, van den Bergh, & Bijlstra, 2020). Yet, some studies have focused on attitudes towards religious minorities (see Rowatt et al., 2005). A Belgian study for example, found that teachers working in schools with a large Muslim student population, have more negative attitudes towards Muslim student in comparison to other teachers (Agirdag et al., 2012).

Dual process models (see for example Fazio, 1990 or Gawronski & Bodenhausen, 2006) state that attitudes can affect people’s behaviour in two ways. First, they can influence behaviour in conscious and deliberate ways, and second, attitudes can be automatically activated and lead to behaviour without conscious deliberation (Gawronski & Bodenhausen, 2006; Nosek, Hawkins, & Frazier, 2011). When attitudes are automatically activated, they can lead to biases in judgement and behaviour that people are unaware of (Greenwald et al., 2002). Implicit attitude measures aim to capture these processes of implicit social cognition. Implicit attitudes measures have the advantage that they are less susceptible to social desirability concerns, which makes them appropriate for the study of sensitive topics such as prejudice and religion. Implicit measures have been shown to be predictive of subsequent interracial and intergroup behaviour, even more so than explicit self‐report measures (Greenwald, Poehlman, Uhlmann, & Banaji, 2009). Likewise, a recent review study on teacher attitudes indicated that teachers’ implicit attitude measures were more predictive of student outcomes than explicit measures (Denessen et al., 2020).

Although implicit measures are increasingly used in educational research (e.g., Kumar, Karabenick, & Burgoon, 2015; Glock, Kneer, & Kovacs, 2013; or for a review see Denessen et al., 2020), most of this research has employed experimental designs and focused judgements of fictional students (e.g., Glock, Beverborg, & Müller, 2016). While those studies have the benefit of control for potential confounding variables, field studies are also needed to examine how teacher attitudes affect outcomes of actual students in educational practice. Yet, research on the associations between implicitly measured teacher attitudes and outcomes of actual students is scarce (for exceptions see van den Bergh, Denessen, Hornstra, Voeten, & Holland, 2010; Hornstra, Denessen, Bakker, van den Bergh, & Voeten, 2010; Peterson, Rubie‐Davies, Osborne, & Sibley, 2016; Thomas, 2017; Bergh et al., 2010). An earlier study on the present data‐set (masked reference) showed that Muslim teachers had a considerably more positive implicitly measured attitude towards Muslims (vs. non‐Muslims) than their non‐Muslim colleagues. Consistent with the research by Towles‐Schwen and Fazio (2006), this difference in attitudes could very well mediate the expected differences in STR quality. Importantly, however, such mediation would be partial, because the aforementioned ‘cultural’ differences could still play a role and affect STR quality regardless of teachers’ religious attitude.

### The present study: Context and hypotheses

In the Netherlands, Islamic education is a form of private education^1^ financed by the government. Although it harbours students from various ethnic and cultural backgrounds, this form of education aims to support the development of students’ Islamic identity and to improve the academic achievement of Muslim students (Shadid & Van Koningsveld, 1995). Dutch Islamic Education is in accordance with the national education curriculum as determined by the Dutch ministry of education, but also offers additional courses such as Islam and Quran education (see Appendix for more information about Dutch Islamic primary schools).

In this study, we examined how primary school students from these Islamic schools perceived the STR. We evaluated six hypotheses. First, we expected that Muslim students would report more closeness (H1), and less conflict (H2) and negative expectations (H3) in the STR if their teacher was Muslim versus non‐Muslim. Additionally, we hypothesized that teachers’ implicitly measured attitude towards Muslims (vs. non‐Muslims) would be positively associated with closeness and negatively with conflict and negative expectations, and – given the difference in this attitude between Muslim and non‐Muslim teachers in our previous study (masked reference) – partly mediate the relationship between teachers’ religious background and students’ perceptions of closeness (H4), conflict (H5), and negative expectations (H6) in the STR. We controlled for children’s gender, grade level, and teachers’ gender and years of teaching experience, as previous studies indicate that boys and older children tend to have less positive relationships with their teachers (Baker, 2006; Spilt, Hughes, Wu, & Kwok, 2012) and that the quality of the STR can vary based on teachers’ gender and experience (Brekelmans, Wubbels, & Van Tartwijk, 2005; Spilt, Koomen, & Jak, 2012).

## Method

### Procedure and participants

In total, 43 Islamic primary schools were approached to participate. At the time of the data collection (spring 2017) there were 49 Islamic primary schools in the Netherlands. Six of these schools had Muslim teachers only and were not approached to take part in this study. Fifteen schools did not respond or declined participation, 14 schools could not be contacted, and four schools agreed to participate but responded too late. Ten schools agreed to participate. After the school boards agreed to participation, teachers were asked for their active consent to participate in the study. Parents received an informed consent letter and had the opportunity to object to participation of their child. The participation rate of the students was 98.3%. Data were collected during school hours. Children filled out the questionnaires anonymously, while teachers filled out a questionnaire on their background characteristics and took an implicit association test (IAT). Children were able to ask one of the researchers for help when they did not understand a question properly.

The final sample consisted of 707 students (56.9% female) from 35 classes (Grade 3 to 6). Students’ mean age was 10.02 years (*SD* = 1.25; range 8–14 years). All students were Muslim but from different ethnic backgrounds. Most children identified themselves as Moroccan (43.0%), Turkish (28.4%), or other (23.5%), 1.4% self‐identified as Dutch, and 3.6% of the students did not report their ethnicity. The 35 participating teachers (85.7% female) had a mean age of 32.94 years (*SD* = 6.37). Twenty teachers identified themselves as Muslim and 15 teachers as non‐Muslim. Most teachers had a Dutch origin (42.9%) of which 57.14% identified themselves as non‐believers, 28.57% as Christian, and 14.28% as Muslim. Followed by Moroccan (28.6% and all Muslim), Turkish (20.0% and all Muslim), and other (8.7%) of which 50% were Christian, 25% Muslim, and 25% had no religion. On average, the teachers had 7.51 years (*SD* = 5.77) teaching experience, and 4.83 years (*SD* = 4.43) in Islamic schools specifically.

### Measures

#### Children’s perception of the STR

The Dutch version of the Student Perception of Relationship with Teacher Scale (SPRTS; Koomen & Jellesma, 2015) was used to assess students’ perceptions of the quality of the relationship with their teacher. In Dutch primary schools, students have typically one or two teachers. If students had multiple teachers, they filled out this scale for the teacher who was present during data collection. The questionnaire consisted of three six‐item subscales for *closeness* (e.g., ‘I feel relaxed with my teacher’), *conflict* (e.g., ‘I easily have quarrels with my teacher’) and *negative expectations* (e.g., ‘I wish my teacher could spend more time with me’.) All items had a five‐point Likert‐type scale with the following response options; 1 (*No!*), 2 (*No, not re*ally), 3 (*Sometimes*), 4 (*Yes, kind of*), and 5 (*Yes!*). Previous research has provided support for the validity and reliability of the measure among a representative sample of primary school students in the Netherlands (Koomen & Jellesma, 2015). A confirmatory factor analysis (CFA) on the data of this study confirmed the three‐factor model, χ^2^ (132) = 388.74, *p* <.001, CFI = .91, TLI = .90, RMSEA = .052. Cronbach’s was α = .84 for closeness, α = .83 for conflict, and α = .69 for negative expectations.

#### Implicitly measured attitude towards Muslims versus non‐Muslims

An IAT was used to measure the strength of teachers’ automatic associations between religious group (i.e., Muslim vs. non‐Muslim) and the valence of words (i.e., positive vs. negative). Prior research has established that IAT measures have satisfactory internal consistency and test‐retest reliability for use in correlational studies (Greenwald & Lai, 2020). In general, IAT’s are also found to have good convergent validity with other implicit measures (Cunningham, Preacher, & Banaji, 2001), and good predictive validity (e.g. Greenwald et al., 2009), although it has been argued that more evidence needed to support their construct validity (Greenwald & Lai, 2020). The present measure was based on an IAT by van den Bergh et al. (2010), which assessed implicit attitudes towards ethnic minorities. In that IAT, teachers were asked to classify names as ‘Dutch’ or Turkish/Moroccan, whereas teachers in this study were asked to classify these names as Muslim or non‐Muslim. The Muslim population in the Netherlands consists almost exclusively of people with an ethnic minority background, mainly from Morocco or Turkey. Therefore, the same names were used in the IAT of this study. Inquisit software (by Millisecond) was used to administer the IAT on a computer or laptop.

The IAT consisted of seven blocks: three practice blocks and four test blocks. Table 1 contains an overview of the Implicit Association test tasks and examples of stimuli.

**Table 1:** Overview of the implicit association tasks

Sequence	Task description
Practice block 1	Classify words as good (e.g., peace) by pressing the E key or bad (e.g., war) by pressing the I key
Practice block 2	Classify names (e.g., Mohammed or Michael) as ‘non‐Muslims’ by pressing the E key or ‘Muslims’ by pressing the I key
Practice block 3 + Test block 4	Name and word categories are paired. When a word or name appeared on the screen, the presence of a positive meaning or a non‐Muslim name had to be responded to by using the E key; the presence of a negative meaning or a Muslim name had to be responded to by using the I key
Practice block 5	Only names had to be classified again but now by using the E key for Muslim names and the I key for non‐Muslim names
Practice block 6 + Test block 7	The word and name categories were paired again but now in the opposite manner: Words with a positive meaning and Muslim names had to be responded to by using the E key, while words with a negative meaning and non‐Muslim names had to be responded to by using the I key

The order of the blocks was randomly counterbalanced across respondents. Response latencies were recorded for each response. In line with Greenwald, Nosek, & Banaji, 2003, we eliminated trials with latencies > 10,000 ms. The response latencies for the blocks in which the participants had to respond similarly to ‘good’ and ‘Muslim’, on the one hand, and ‘bad’ and ‘Non‐Muslim’, on the other hand, were then compared with the response latencies of blocks in which the participants had to respond similarly to ‘bad’ and ‘Muslim’, on the one hand, and ‘good’ and ‘non‐Muslim’, on the other hand. The underlying assumption is that greater difficulties with the association of two particular categories (i.e., ‘good’ and ‘Muslim’) will produce longer response times for these pairs when compared with other pairs. In line with Greenwald, McGhee, and Schwartz (1998), the error rate was about 4%. No participants had to be removed because of excessively high error rates or because of extremely short latencies (>10% of trials with a latency < 300 ms). Trials with latencies > 10,000 ms were eliminated. The scores were calculated using the improved scoring algorithm of Greenwald et al. (2003), by calculating the mean response latency of each block and computing the difference between the blocks with the different pairings of target and evaluations and dividing the difference by the pooled standard deviation of the participant. The standardized score (*D*) was then taken to be an indicator of a teacher’s implicit attitude towards Muslims versus non‐Muslims. Higher scores indicated a greater preference for Muslims over non‐Muslims. The internal consistency of this IAT, calculated by the method described by Bosson, Swann, and Pennebaker (2000), was α = .74 which indicates good reliability.

### Data analysis

To take the hierarchical structure of the data into account (students nested in classes), multilevel analyses were performed in MPlus (Version 8.2; Muthén & Muthén, 2018). All hypotheses were tested at once by specifying a multivariate multilevel mediation model (path model), which included *teacher religion* as independent variable, *closeness*, *conflict*, and *negative expectations* as dependent variables, and the *implicit attitude* of the teacher as mediator. Teachers’ gender, age, years of teaching experience, and years of experience in Islamic schools, and students’ gender and grade (school year) were taken into account as covariates. There were no missing values for the teacher data. There were few missing data at the student level (<2.5%), which were handled by the full information maximum‐likelihood (FIML) method (Schafer & Graham, 2002). The analyses were therefore performed with MLR (maximum likelihood estimation with robust standard errors) as estimator to account for non‐normality.

The multilevel model included a *between* (class) level and a *within* (student) level. The relations of interest were all situated at the between level, as the independent variable and mediating variable (teacher religion and implicitly measured attitude) were assessed at the class level and contained no variance at the within level. Likewise, the covariates teachers’ gender, age, years of teaching experience, years of experience in Islamic schools, and students’ grade were between level variables. At the within level, student gender was included as a covariate. All categorical predictors were entered as dummy variables. Continuous variables were grand‐mean centreed before they were entered in the models. In a first step, all predictors, including the covariates, were included in the model, Next, non‐significant relations were set to zero to obtain the most parsimonious models (Kline, 2005). The significance of indirect paths was tested using a bootstrapping re‐sampling procedure (*N* = 1,000).

The significance of the coefficients for the different predictor variables was tested using Wald tests (*z* tests). The set level of significance was 5%. Model fit was evaluated based on the Akaike Information Criterion (AIC), root‐mean‐square error of approximation (RMSEA), the comparative fit index (CFI), and the chi‐square. Smaller values of the AIC indicate better fit, an RMSEA below.05 indicates good fit of a model, and values between.05 and.08 indicate reasonable fit. Values above.10 indicate poor fit. A CFI above .90 indicates acceptable fit and above.95 indicates good fit of a model (Hu & Bentler, 1999; Kline, 2015).

## Results

### Preliminary analyses

Although the focus of this study is on religion rather than ethnicity, we explored whether the TSR differed between students with different ethnic background, by comparing the two largest ethnic groups, that is, students with Turkish and Moroccan backgrounds. The other groups were too small to include in this comparison. Results of *t*‐tests indicated that students with Turkish and Moroccan backgrounds did not differ significantly on closeness (*t*(501) = 0.214, *p* = .831) or conflict (*t*(460.12) = −1.70, *p* = .090)), but there was a significant difference in negative expectations, *t*(498) = 2.71, *p* = .006, with higher scores for Turkish versus Moroccan students (*M* = 2.33, *SD =* .82; vs. *M* = 2.12, *SD =* .89). Therefore, student ethnicity was added as a covariate (dummies) in subsequent analyses. In addition, we tested whether students’ STR reports differed based on whether they had the same ethnicity as their teacher. Most students had a different ethnicity than their teacher (*N* = 493; 73.5%) and for *N* = 178 students (26.4%) there was an ethnic match. Ethnic match (yes/no) was not significantly related to TSR quality.

### Descriptive statistics and correlations

Table 2 contains the descriptive statistics and intercorrelations for the main variables. On average, students reported a comparatively high level of closeness and low level of conflict. There were no significant differences in TSR quality for students with a Muslim or non‐Muslim teacher. The implicitly measured attitude of Muslim teachers was more positive than that of non‐Muslim teachers, *t*(19.85) = −3.81, *p* = .005, *d* = 1.13. For the Muslim teachers, it was significantly higher than zero, *t*(19) = 6.12, *p <*.001, suggesting a preference for Muslim over non‐Muslims, but for the non‐Muslim teachers, it did not differ significantly from zero, *t*(14) = −0.70, *p* = .497, suggesting a neutral attitude. Table 2 also reports the intraclass correlations (ICCs) of the dependent variables, which indicate that between 6% and 14% of the variance in the STR was situated at the class level, while most variance was situated within classes.

**Table 2:** Descriptive statistics and intercorrelations

	Total						Muslim teachers	Non‐muslim teachers	*t* (*df*)	*p*
	*N*	1	2	3	*M* (SD)	ICC	*M* (*SD*)	*M* (*SD*)		
1. Closeness	704				3.84 (0.91)	0.09	3.82 (0.90)	3.86 (0.93)	0.66 (702)	.512
2. Conflict	700	−0.60**			1.78 (0.88)	0.06	1.74 (0.79)	1.84 (0.98)	1.41 (525.49)	.158
3. Negative expectations	702	−0.29**	0.47**		2.18 (0.86)	0.14	2.19 (0.86)	2.17 (0.86)	‐0.32 (700)	.750
4. IAT Muslims	35	−0.11	−0.19	0.10	0.21 (0.55)	‐	0.46 (0.33)	−0.11 (0.63)	‐3.18** (19.85)	.005

**p < .01.

### Multilevel model

**Figure 1:**
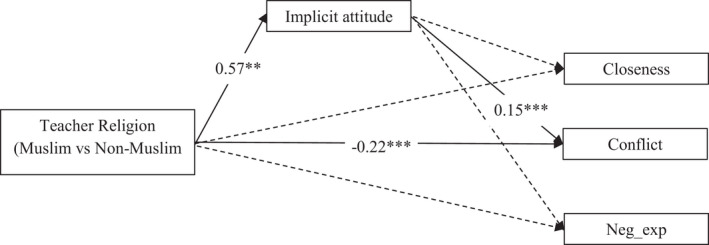
Multilevel path model predicting closeness, conflict, and negative expectations, from teachers’ religion and implicit attitudes (unstandardized estimates at the between level). *Note:* Non‐significant paths, covariates, error terms, and covariances are not depicted

A multilevel model was specified to test the hypotheses simultaneously. This model fitted the data well, *X* ^2^(23) = 47.777, *p* = .002; AIC = 5049.603; RMSEA = .039; CFI = .960. Results are shown in Table 3 and Figure 1. Non‐significant relations were set to zero to reach a parsimonious model. Figure 1 depicts the between level relations.

**Table 3:** Results of multilevel model predicting closeness, conflict, and negative expectations, from teachers’ religion and implicit attitudes (unstandardized and standardized estimates)

	Implicit attitude			Closeness			Conflict			Negative expectations		
	*B*	*SE*	β	*B*	*SE*	β	*B*	*SE*	β	*B*	*SE*	β
Within level												
Covariates												
Student gender (girl = 1, boy = 0)	–			.26**	.09	.15	−.21*	.08	−.12	.27***	.06	0.17
Ethnicity Moroccan (ref =Turkish)										−.14**	.05	−0.09
Ethnicity Other (ref =Turkish)										.26	.33	0.04
Between level												
Covariates												
Grade (year)	ns			ns			ns			−.16***	.03	‐0.63
Teacher gender (female = 1, male = 0)	ns			.35***	.06	.45	ns			ns		
Teaching experience	ns			ns			.01*	.004	.31	ns		
Direct effects												
Teacher religion (Muslim = 1, non‐Muslim = 0)	.57**	.17	.52	ns			−.22***	.05	−.62	ns		
Implicitly measured attitude	‐			ns			.15***	.03	.46	ns		
Indirect effects												
Teacher religion > Implicit attitude > Conflict	‐			ns			.09*	.03	.24	ns		
Total effects												
Teacher religion (Muslim = 1, non‐Muslim = 0)							−.14*	.06	−.38			
Variance components												
*R* ^2^ Level 2	.27			.20			.39			.39		
*R* ^2^ Level 1	‐			.02			.01			.04		

Note.

ns = not significant at *p* < .05.

*p < .05, **p < .01, ***p < .001.

Overall, we obtained little support for our hypotheses. Teacher religion was not significantly associated with closeness (H1) and negative expectations (H3) in the STR, and the indirect effects of teachers’ religion on both variables (H4 and H6) via their implicitly measured attitude were not significant. Still, in line with H2, a significant total effect was found of teacher religion on conflict, *b* = −.14, *p* = .028, indicating that students of Muslim teachers reported less conflict compared with students of non‐Muslim teachers, when the other effects were controlled for. The corresponding standardized coefficient indicated a medium‐sized effect for this difference, b* = −.38. The indirect relation between teacher religion and conflict via teachers’ implicitly measured attitude was statistically significant. However, contrary to our expectations (H5), this effect was positive, as teachers’ implicitly measured attitudes towards Muslim children were related to more conflict. Moreover, the direct effect of teacher religion on conflict was significant and negative. Together, these findings indicate that teacher religion had two opposite effects that counteracted each other, resulting in a small total effect. Additionally, we tested a model in which we excluded implicitly measured attitudes. In this model, there was no significant relation between teacher religion and conflict, *b* = −.07, *p* = .395.^1^

With regard to the covariates (not shown in the figure), it was found that female students reported more closeness, less conflict, and more negative expectations compared with male students. Additionally, it was found that students in higher grades reported less negative expectations. Students furthermore reported more closeness with female teachers compared with male teachers and less conflict with more experienced teachers.

## Discussion

Prior research has shown that minority students can be at risk for developing less favourable affective relationships with their teachers (Clewell & Villegas, 1998; Irvine & Fenwick, 2011; Maylor, 2009; Naman, 2009; Spilt & Hughes, 2015), especially when those relationships are ethnically or racially incongruent (Rasheed et al., 2019; Thijs et al., 2012). Religious minority students are also likely to be in religiously incongruent STRs. However, in the Netherlands, an increasing number of Muslim children attends Islamic schools with both Muslim and non‐Muslim teachers (Driessen & Valkenberg, 2000; Dronkers, 2016). Thus, the context of Islamic education offers a unique opportunity to compare religiously congruent and incongruent STRs. The present research contributes to the literature by making this comparison.

We examined three aspects of student‐reported STR quality (closeness, conflict, and negative expectations) in Islamic primary school children, and we tested whether their teacher’s implicitly measured attitude towards Muslims (vs. non‐Muslims) mediated the relations between the teacher’s religious background and these three relationship aspects. We expected that the quality of the perceived STR would be better for Muslim students with a Muslim versus a non‐Muslim teacher. This was the case for conflict, but not for closeness or negative expectations. Consistent with previous findings on ethnically incongruent STRs (e.g., Thijs et al., 2012), conflict was higher in religiously incongruent ones, but only in our regression model which included teachers’ implicitly measured attitude towards Muslims (vs. non‐Muslims) and the control variables. That is to say, there were no simple mean differences (*t* tests) in relationship quality with Muslim versus non‐Muslim teachers. Contrary to expectations, teachers’ attitude was related to more rather than less conflict – something that we will further discuss below – and this relation suppressed the direct effect of teacher religion. Thus, the lower rates of conflict in religiously congruent STRs emerged under the rather unlikely condition that Muslim and non‐Muslim teachers had a similar attitude.

It is not fully clear why the effect of teacher religion was obtained for conflict only. Perhaps the absence of effects for closeness and negative expectations can be explained by the possibility that, more so than conflict, those relationship dimensions reflect the perceived emotional availability of the teacher. Thus, both relationship aspects might be primarily determined by the sensitivity of the teacher, and this sensitivity might be relatively independent of differences in cultural‐religious background (see Mesman, 2018, for a debate on this in the attachment literature). Although the distrust that is typical for relational conflict could indicate emotional insecurity, this relationship dimension could also reflect more ‘mundane’ irritations (e.g., ‘I easily have quarrels with my teacher’) resulting from cultural misunderstandings and miscommunications. Of course, future research is needed to examine these *post‐hoc* interpretations.

Additionally, it is not clear how the effect of teachers’ implicitly measured attitude on conflict should be explained. Bonefeld and Dickhäuser (2018) found that teachers with more positive implicit attitudes towards migrant students tended to grade those students more poorly than teachers with more negative attitudes. This was explained by the possibility that positive implicit attitudes might have evoked high academic and behavioural expectations that could not be met. A similar explanation might account for this unexpected finding in this study. It could be that teachers with a very positive attitude towards Muslims might hold very high expectations of Muslim students, which might be difficult to meet and thus cause negativity and conflict in the STR. Indeed, research on parental expectations indicates that unrealistic expectations can cause disappointment and poor relationships (Russel, 2003). Still, this interpretation is mere speculation, and there may be other explanations for the unexpected finding for conflict. It is important to note, for example, that there were no extremely negative attitudes towards Muslims in the teacher sample. The Muslim teachers showed a clear preference for their in‐group, and the attitude of their non‐Muslim colleagues was neutral indicating a potential selection effect of working in Islamic education. However, research in other contexts typically finds that teacher attitudes towards minority groups tend to be rather negative on average (see Pit‐Ten Cate & Glock, 2019, for a meta‐analysis), and such negative attitudes may have harmful effects for minority students (e.g., Peterson et al., 2016; Thomas, 2017; van den Bergh et al., 2010). For future research, it would therefore be interesting to also include non‐Muslim teachers who teach Muslim students at non‐Islamic schools. Presumably, these teachers would have less positive attitudes towards Muslims compared with (Muslim and non‐Muslim) teachers working at Islamic schools.

All in all, our findings provide only moderate support for the notion that religious congruence contributes to the quality of the STR in Islamic schools. Apparently, there is slightly less risk for conflict in congruent relationships, but this benefit can be offset if the teacher has a very positive attitude towards the student’s religious group. Yet despite the potential disadvantage of such a positive attitude, the children in our study seemed to experience high‐quality relations (close and non‐conflictual) with their teacher, regardless of religious background. Moreover, although we did not use the exact same items, these relationship reports seem to be better than those obtained in similar research among ethnic minority students (mostly Turkish and Moroccan) at regular primary schools (e.g., De Jong et al., 2018). This suggests that Islamic schools may indeed provide a ‘safe haven’ for Muslim children (Driessen & Merry, 2006) and that having a religious heterogeneous workforce at Islamic schools does not have a harmful impact on the students.

### Limitations and strengths

In further evaluating the present findings, some other qualifications need to be considered. First, our research was conducted in the specific context of Islamic education, which means that its findings cannot be directly generalized to other contexts. Particularly the link between teachers’ implicitly measured group attitudes and STR quality needs further investigation in schools where the group identity of minority students is less strongly supported. Moreover, religious incongruence might have different effects for students from other religious backgrounds. Still, due to their increasing presence in the educational landscape, it is important to include Western Islamic schools in educational research, and our study was one of the very few to do so.

Next, most attitude models (e.g., Fazio, 1990; Gawronski & Bodenhausen, 2006) as well as empirical studies (Denessen et al., 2020) have focused on how implicitly measured attitudes affect the judgements or behaviours of the persons holding them. Instead, we used a more stringent test of their effects by including student perceptions as outcome measures. This approach can be considered a strength of our study, but it also assumes that the link between teachers’ implicitly measured attitudes and student‐perceived STR quality can be explained by teachers’ behaviours towards their students. We could not test this assumption, which means, again, that the unexpected effect of conflict should be interpreted with care. Future studies should therefore examine the actual interactions between teachers and students, or ask teachers about the STR.

Another limitation is that our study has a cross‐sectional design. This means that we cannot draw causal conclusions. Still, teacher’s religion clearly was an independent variable, and it is difficult to see why relational conflict with Muslim students would increase rather than diminish positivity towards Muslims.

Fourth, we did not include any explicit attitude measures. Despite evidence that implicit attitude measures are generally more predictive of subsequent interracial and intergroup behaviour than explicit ones (Greenwald et al., 2009), it would have been valuable to use both measures.

Lastly, religion and ethnic background were confounded in this study. Thus, we cannot fully exclude the possibility that the effects of teacher religion may be partly due to teachers’ ethnicity. As there are few Muslim teachers in the Dutch teacher force, let alone native Dutch Muslim teachers, it is not possible to disentangle these effects. Likewise, although the implicit attitude measures targeted attitudes towards Muslims versus non‐Muslims (by using those categories in the IAT), teachers’ implicit attitudes toward Muslim students’ may be confounded with their attitudes towards ethnic minority students as most Muslim students also have an ethnic minority background. By combining implicit measures towards religious and ethnic minority groups, future research could examine the possible overlap between both types of attitude targets. Despite its shortcomings, our research has two strengths beyond those already mentioned. To our knowledge, it is the first study to examine affective STRs in the context of religious instead of ethnic differences. In addition to this, it responds to the need for more studies on the effects of implicitly measured teacher attitudes on real students, rather than hypothetical ones in experimental or vignette research (Denessen et al., 2020).

### Conclusion

Overall, the findings of this study indicate that religious incongruence does not play a major role in STR quality in Islamic primary schools. We did find that students with a non‐Muslim teacher reported more conflict in comparison to students with a Muslim teacher. Yet although this effect was medium in size, it was not complemented by differences in closeness and negative expectations. Still, future studies could examine the impact of religiously incongruent STRs for students who are religious minorities in their schools, and further consider the role of the religious attitudes of their teacher. We hope this study inspires such research.

### Notes

We also explored the interaction effects of teacher religion and implicitly measured attitudes on the three relationship aspects. None of these interactions were significant.

## Conflicts of interest

We have no known conflict of interest to disclose.

## Author contributions

**Fatima Zohra Charki:** Conceptualization (equal); Data curation (equal); Formal analysis (equal); Investigation (equal); Methodology (equal); Project administration (equal); Writing – original draft (equal); Writing – review & editing (equal). **Lisette Hornstra:** Conceptualization (equal); Formal analysis (equal); Methodology (equal); Supervision (equal); Writing – review & editing (equal). **Jochem Thijs:** Conceptualization (equal); Methodology (equal); Supervision (equal); Writing – review & editing (equal).

## Data Availability

The data of this study will be made available in a public archive.

## Footnotes

^1^
Public schools are administered under the auspices of the community government, whereas private schools (which are almost all denominational schools) are administered by private legal institutions (usually a foundation).

## Appendix

The first Islamic primary schools were founded in the early 1980s, mainly by Moroccan and Turkish migrants. Dissatisfaction of Muslim parents regarding public education, such as a lack of Islamic instruction and poor academic performance of their children, was a main reason and motive for the foundation of Islamic schools (Driessen & Valkenberg, 2000). Islamic primary education is a form of private education in the Netherlands financed by the government. The Dutch constitution allows the foundation and organization of regular education based on religious or philosophical grounds (Driessen & Merry, 2006). Thereby, Islamic schools, as well as other religious schools, receive the same funding from the government as public schools. Islamic schools teach in accordance with the national education curriculum as determined by the ministry of education. Additionally, they offer courses such as Islam and Quran education. Religion forms the thread that determines expected habits and behaviour of teachers and students. For example, Islamic schools only celebrate holidays that are related to the religion such as Eidu lfitr
^2^
Islamic holiday at the end of the fasting month Ramadan. and Eidu l‐adha
^3^
Festival of the sacrifice. and they set aside time for teachers and students to perform their daily prayers (Driessen & Valkenberg, 2000).

The aims of Islamic primary education are: (1) Supporting the development of the cultural and Islamic identity of students and (2) improving the academic achievement of Muslim students (Shadid & Van Koningsveld, 1995). Islamic schools aim to be a safe haven for their pupils and offer them the opportunity to improve their academic achievement (Driessen & Merry, 2006). The majority of the students in Dutch Islamic schools are from Moroccan or Turkish origin and are all Muslim (Driessen, 2008). Not all teachers at these schools are Muslims themselves (Dronkers, 2016) and although recent information on the religious background of teachers in Islamic schools is not available, a study from 2000 showed that a considerable portion (approximately 70%) of the teachers had a non‐Islamic background (Driessen & Valkenberg, 2000).
